# Small-Pox Hospital for Barnsley

**Published:** 1898-08-13

**Authors:** 


### Small-pox Hospital for Barnsley.

Barnsley is about to acquire a small-pox hospital to serve for the surrounding districts also. Hitherto small-pox cases have been received into the present isolation hospital at Kendray, which was established through private charity. Recently the accommodation has proved inadequate, and the local authorities being appealed to, they have elected to provide a new hospital for small-pox only, rather than increase the accommodation for infectious diseases generally. The site for the new building is on a hill, quite apart from other buildings, and occupies land unlikely to be built on at present, two miles from the centre of Barnsley. The hospital will comprise a group of five blocks, consisting of an isolation block, with male and female wards, nurses' duty rooms and offices, an administrative block, entrance and discharge block, and laundry and disinfecting block. The principal wards are to be 36 ft. by 26 ft., and two smaller wards are provided for special cases. The walls are to be of red brick with stone mouldings and dressings. The cost is estimated at about £6,000, and the site cost £1,000.

